# Obituary, Dr. M. E. Potter

**Published:** 1875-11

**Authors:** 


### OBITUARY.

Died, at his residence in Attica, Wyoming Co., N. Y., on Monday, November 1st, 1875, Dr. M· E. Potter, aged sixty.

Dr. Potter began the practice of medicine at the early age of twenty, having been licensed by the Genesee Co., Medical Society after attending two courses of lectures at the Geneva Medical College. He began the practice of medicine in Bennington Centre, Wyoming Co., where he remained for eleven years; from thence he removed to Cowlesville, where he practiced for nineteen years, the balance of his life being passed in Attica. After having practiced for a few years, and having attained his majority, he returned to the Geneva College and obtained his diploma, Dr. Potter was a member of the Genesee County Medical Society, and of the Wyoming County Society since its foundation, a few years ago. For a number ot years he was one of the Curators of the Buffalo College, and almost always made it a point to be present at the Commencement exercises. Throughout the entire course of his professional career he was largely engaged in the practice of medicine, a large amount of which was consultation practice. His ambition was to be actively engaged in practice, and the circumstances of the patient seldom, if ever, had any influence on the promptness of his attentions. Almost his last visit was made to a poor man who informed him in advance that he could not pay for his services.

Dr. Potter left three children, two daughters, and a son, Milton G. Potter, M. D., Prof. of Anatomy in the Buffalo Medical College,

His funeral was attended from his late residence, on Wednesday, Nov. 3d, and was largely attended by neighboring practitioners. The cause of his death was a serous effusion at the base of the brain.

He will long be mourned by a wide circle of friends and patients. Our deepest sympathy is extended to those who by his death are bereft of a kind parent and a trusted counselor.

